# Clinical manifestations of new *versus* recrudescent malaria infections following anti-malarial drug treatment

**DOI:** 10.1186/1475-2875-11-207

**Published:** 2012-06-18

**Authors:** Ayesha M Shaukat, Elizabeth A Gilliams, Leo J Kenefic, Matthew B Laurens, Fraction K Dzinjalamala, Osward M Nyirenda, Phillip C Thesing, Christopher G Jacob, Malcolm E Molyneux, Terrie E Taylor, Christopher V Plowe, Miriam K Laufer

**Affiliations:** 1 Malaria Group, Howard Hughes Medical Institute/Center for Vaccine Development, University of Maryland School of Medicine, Baltimore, MD, USA; 2 Blantyre Malaria Project, University of Malawi College of Medicine, Blantyre, Malawi; 3 Malawi-Liverpool-Wellcome Trust Clinical Research Programme, College of Medicine, University of Malawi, Blantyre, Malawi; 4 Department of Medicine, College of Osteopathic Medicine, Michigan State University, East Lansing, MI, USA; 5 Liverpool School of Tropical Medicine, University of Liverpool, Liverpool, UK

**Keywords:** Malaria, Sulphadoxine-pyrimethamine, Drug efficacy, Genotyping, Recrudescent infections, New infections, Malawi, Anaemia

## Abstract

**Background:**

Distinguishing new from recrudescent infections in post-treatment episodes of malaria is standard in anti-malarial drug efficacy trials. New infections are not considered malaria treatment failures and as a result, the prevention of subsequent episodes of malaria infection is not reported as a study outcome. However, in moderate and high transmission settings, new infections are common and the ability of a short-acting medication to cure an initial infection may be outweighed by its inability to prevent the next imminent infection. The clinical benefit of preventing new infections has never been compared to that of curing the initial infection.

**Methods:**

Children enrolled in a sulphadoxine-pyrimethamine efficacy study in Blantyre, Malawi from 1998–2004 were prospectively evaluated. Six neutral microsatellites were used to classify new and recrudescent infections in children aged less than 10 years with recurrent malaria infections. Children from the study who did not experience recurrent parasitaemia comprised the baseline group. The odds of fever and anaemia, the rate of haemoglobin recovery and time to recurrence were compared among the groups.

**Results:**

Fever and anemia were more common among children with parasitaemia compared to those who remained infection-free throughout the study period. When comparing recrudescent vs. new infections, the incidence of fever was not statistically different. However, children with recrudescent infections had a less robust haematological recovery and also experienced recurrence sooner than those whose infection was classified as new.

**Conclusions:**

The results of this study confirm the paramount importance of providing curative treatment for all malaria infections. Although new and recrudescent infections caused febrile illnesses at a similar rate, recurrence due to recrudescent infection did have a worsened haemological outcome than recurrence due to new infections. Local decision-makers should take into account the results of genotyping to distinguish new from recrudescent infections when determining treatment policy on a population level. It is appropriate to weigh recrudescent malaria more heavily than new infection in assessing treatment efficacy.

## Background

Anti-malarial drug efficacy studies serve several purposes. They are used to assess drug efficacy and safety for licensing, for comparison of efficacy across different settings, and sometimes for validation of markers of drug resistance. For any of these purposes, controlling for differences in malaria transmission intensity is essential in drug efficacy studies. Episodes of recurrent parasitaemia following treatment may be due to recrudescence of the initial infection, reflecting failure of the drug to clear the infection; or, they may be due to new infections that occurred during the follow-up period. Genotyping of polymorphic genes or short tandem repeats (microsatellites) is used to distinguish recrudescent from new infections. New infections are not considered a failure of the curative efficacy of the drug. This practice of polymerase chain reaction (PCR) correction of efficacy, however, leads to the discounting of the post-treatment prophylactic efficacy that can be an important secondary benefit of drug treatment, especially with longer-acting anti-malarial drugs. The ability of a drug to prevent new infections for weeks or even months after an acute treatment episode is not taken into account when measuring efficacy in the standard way. A drug that appears to cure the initial infection but offers no protection against the next infection may appear to have the greatest clinical success rate, but in higher transmission settings, new infections are common and the ability of a short-acting medication to cure an initial infection may be outweighed by its inability to prevent the next imminent infection.

One way to determine the relative importance of preventing new versus recrudescent infections is to compare the clinical burden of each of these types of infection. If one type is more likely to cause fever (the most common clinical manifestation of malaria) or increase the risk of anaemia, then it may be possible to determine if preventing recrudescent infections is as important as curing the original one. In a meta-analysis conducted by Olliaro and colleagues, the results of 13 studies in Africa across different countries and using different treatments showed that individuals with new infections developed fever sooner than those who experienced recrudescent infections, although the risk was high in both groups [1]. However, the findings among the different studies were heterogeneous and when multivariate analysis was performed, risk of fever was only associated with the parasite density of the recurrent infection and was inversely related to age. The authors did not review the effect on haemoglobin concentration, although anaemia is one of the common morbidities associated with malaria.

Long-standing surveillance of antimalarial drug efficacy in a stable population provides a unique opportunity to compare the clinical presentation of new and recrudescent infections. The efficacy of sulphadoxine-pyrimethamine (SP) was monitored over a six-year period in a large cohort of children in Malawi, an area of moderate perennial malaria transmission. The study took place towards the end of the period when SP was Malawi's national first-line treatment for uncomplicated malaria, when SP was modestly efficacious, and when both new and recrudescent infections were expected to occur.

## Methods

### Data collection

Blood samples were obtained for this study from February 1998 to June 2004 at the Ndirande Health Centre in Blantyre, Malawi [2]. Study participants were children aged five months through 10 years who had symptoms of malaria and *Plasmodium falciparum* detected in their peripheral blood smear by microscopy. The day of study enrolment was designated day 0 and participants were given standard treatment doses of SP (500 mg sulphadoxine and 25 mg pyrimethamine per tablet, ¼ tablet per 5 kg weight). Follow-up took place on days 1, 2, 3, 7, 14, and 28 and any time a participant was ill prior to day 28. At each visit, a malaria smear was obtained, haemoglobin concentration was measured (HemoCue Angelholm, Sweden) and drops of blood were collected on filter paper. Specimens from participants who were found to have recurrent parasitaemia from day 14 through 28 underwent genotyping as described below. Children who were febrile at the time of recurrent parasitaemia were treated with halofantrine rescue therapy and terminated from follow-up. Participants with asymptomatic infections were followed according to the study schedule and were provided rescue therapy when they developed fever or reached day 28. Participants who were successfully treated and did not experience recurrent infection through day 28 were included as the baseline group. The study protocol was approved by the University of Malawi College of Medicine Research and Ethics Committee and the University of Maryland Baltimore Institutional Review Board.

DNA was extracted from the dried blood spots on filter papers using the Qiagen BioRobot Universal System with the QIAamp Investigator BioRobot Kit (Germantown, MD, USA) for day 0 and the day(s) of recurrent parasitaemia. Based on previous studies within the authors’ research group, the six microsatellites with the greatest genetic diversity among the 12 neutral microsatellites, that have been reported previously to adequately discriminate among parasites from a variety of settings, were used [3]. The markers Poly alpha, PfPK2, TA81, ARA2, TA87, and TA40 were amplified using a hemi-nested PCR approach, described in detail on the referenced website [4]. Samples were processed for capillary electrophoresis using an Applied Biosystems 3730XL high-throughput 96-capillary DNA sequencer and software (Foster City CA, USA).

### Microsatellite analysis

Capillary electrophoresis output files were analysed using Genemapper 4.0 (Applied Biosystems, Foster City CA, USA) with default microsatellite analyses settings. Genomic control strains, 3D7 and HB3 (ATCC-MR4, Manassas VA, USA) were included to determine the characteristic morphology of peaks for each marker and to control for slight variations in capillary electrophoresis. Only peaks with the characteristic morphology above the threshold of 100 relative fluorescent units were scored and companion peaks were omitted. Alleles were sized by manual inspection of each electropherogram and then normalized against the 3D7 control. The adjusted peak sizes were rounded to the nearest three nucleotide repeat size using a custom Perl script. Samples were analysed once the data had been adjusted and binned. Details about the PCR and scoring protocol are published on the investigators’ website [4].

### Genotype classification

Parasite genotypes present on the day of recurrent malaria infection were compared to genotypes present on day 0. Samples with detectable alleles for five or six microsatellite loci were considered complete and were classified as new, recrudescent or uninterpretable. A recrudescent infection had matching alleles at every locus or all but one locus when comparing genotypes on day 0 and day of recurrent parasitaemia. We allowed for the absence of a matching allele at one locus because with polyclonal infections, alleles may occasionally be undetectable [5]. A new infection had ≤3 microsatellite loci with matching alleles between day 0 and the day of recurrent parasitaemia. Uninterpretable samples had four out of six microsatellite loci with matching alleles between day 0 and day of recurrent parasitaemia. Multiplicity of infection (MOI) was defined as the highest number of alleles found at a single locus and represents the number of simultaneous infections carried in a patient.

### Clinical definitions

Fever was defined as an axillary temperature ≥37.5°C at the time of evaluation at the study clinic. Mild anaemia was defined as a haemoglobin concentration <11.0 g/dL and moderate anaemia as a haemoglobin level of <7.0 g/dL. Haematological recovery was calculated by subtracting the day 0 haemoglobin concentration from the haemoglobin concentration on the day of recurrent infection.

### Statistical analysis

Data were analysed in STATA 12.0 (StataCorp, College Station, TX, USA). Baseline characteristics including age, geometric mean parasite density and haemoglobin concentration among participants who experienced no recurrent infection, recrudescent infection and new infections were compared by ANOVA. MOI was only measured in samples from participants with recurrent infection. To assess clinical outcome at the time of recurrence or at the end of the study for participants who did not experience recurrent infection, incidence of fever and anaemia, mean haemoglobin and mean change in haemoglobin from baseline were compared between the three groups. The relationship between recurrent infection status and these key clinical outcomes were assessed using logistic and linear regression, using participants without recurrent infection as the referent. Logistic regression and Student’s *t*-test were used to compare odds ratios and means between new and recrudescent infections. Multivariate logistic regression was used to assess the relationship of each clinical factor to new compared to recrudescent infection. In the multivariate regression, year was included in addition to the other variables analysed individually. When more than one episode of recurrent parasitaemia occurred in an individual, each pair from day 0 and the day of failure was treated independently and the Huber/White/sandwich estimator of variance was used to control for non-independence of measures within a single individual. The analyses were subsequently repeated using only the first or only the final episode of parasitaemia for individuals with more than one episode or recurrent infection. Kaplan-Meier curves to analyse time to first recurrent infection and symptomatic infection by recurrent infection classification were compared using the log rank test.

## Results

Over the six-year study period, 279 participants were followed for 28 days with no recurrent infection and 265 had episodes of recurrent parasitaemia between days 14 and 28. Microsatellites were amplified from initial and recurrent infections in 222 pairs of infections from 195 children. The infections were classified as 119 recrudescent infections, 84 new infections and 19 with uninterpretable results. Baseline characteristics of the three groups are presented in Table 1. There was no difference in age between the children who had no recurrent infection, recrudescent infection or new infection. Initial parasite density and haemoglobin concentration did not differ between subjects who later developed new compared to recrudescent infections (p = .10 and p = .15, respectively).

**Table 1:** Baseline characteristics of participants who were successfully treated for their infection and those that experienced recrudescent or new infections: mean (95% confidence interval)

	**No recurrent infection**	**Recrudescent**	**New infection**	**P value**
Age in months	33.9 (30.6-37.2)	33.0 (28.7-37.3)	35.7 (29.5-41.9)	.8
Parasites/mm^3^	7,365 (5408–10,029)	26,291 (17,508-39,480)	15,331 (9,164-25,649)	.002
Haemoglobin in gm/dL	9.8 (9.6-10.1)	9.3 (9.0-9.6)	9.0 (8.5-9.4)	.009
Multiplicity of infection	Not available	3.1 (2.8-3.5)	2.5 (2.3-2.9)	.03

### Clinical symptoms in patients with recrudescence *versus* reinfection

Temperature was recorded for all participants at the time of recurrent infection and for 277/279 of the participants who had successful treatment. Measurement of haemoglobin at the time of recurrent parasitaemia or the end of the study was available for all but three of the participants.

Table 2 shows the prevalence of fever and anaemia and the mean haematological recovery among children who experienced no recurrent malaria, recrudescent infection and new infection. Fever and anaemia were more common among children with parasitaemia compared to those without infection. The haematological recovery did not differ between the groups when measured in univariate analysis. Only five patients had moderate anaemia on the day of recurrent parasitaemia and they were equally distributed among recrudescent and new infections (two and three, respectively).

**Table 2:** Clinical characteristics at the time of recrudescent and new infection compared to participants at the end of the study without recurrent infection

	**Distribution**	**Odds ratio (95% CI) univariate**	**P value**	**Odds ratio (95% CI) Multivariate Recrudescent vs. new**	**P value**
	**Proportion (%)**				
Fever					
No	11/277(3.9)	Referent			
Recrudescent	26/119 (21.8)	6.8 (3.2-14.3)	<.001	Referent	
New	13/84 (15.4)	4.4 (1.9-10.3)	.001	.6 (.2-1.5)	.27
Anaemia					
No	137/275 (49.8)	Referent			
Recrudescent	81/117 (69.2)	2.3 (1.4-3.7)	.001	Referent	
New	54/83 (65.1)	1.9 (1.1-3.2)	.017	1.5 (.7-3.2)	.34
	Mean (95% CI)				
Change in haemoglobin					
No	.9 (.8-1.2)				
Recrudescent	.7 (.4-1.0)		.2	Referent	
New	1.2 (.7-1.6)		.4	1.3 (1.1-1.6)	.01
Multiplicity of infection					
No	N/A				
Recrudescent	2.7 (2.3-3.0)			Referent	
New	2.3 (2.1-2.6)		.1	.8 (.6-1.0)	.05

In multivariate analysis, fever and the presence of mild anaemia were not associated with the classification of new vs. recrudescent infection. However, children with new infection had an improved haematological recovery compared to those who experienced recrudescent infections, after controlling for other confounding variables.

**Figure 1:**
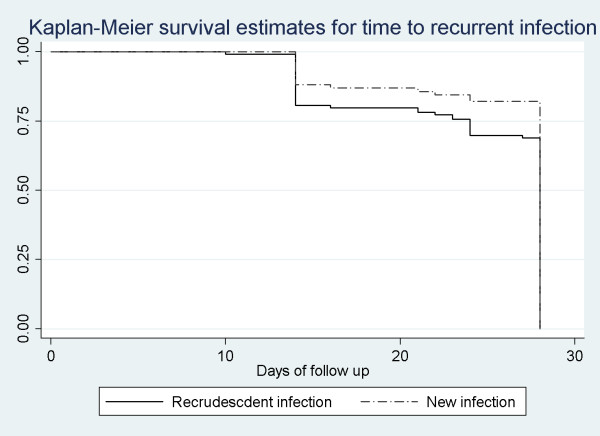
**Kaplan-Meier survival curves for time to recurrent infection, comparing new versus recrudescent infections**.

First parasitemia occurred sooner for recrudescent compared to new infection (Figure 1, p = .03). The time to febrile parasitemia was the same for both groups (p = .14).

Twenty-one participants had two or three time points when recurrent parasitaemia was detected and had interpretable molecular results. Twelve children had repeated episodes of recrudescent infections, five had repeated episodes of new infections, three had new followed by recrudescent infections and one had a recrudescent followed by a new infection. As a sensitivity analysis, the analyses were repeated including only the first recurrent infection and again including only the final recurrent infection. The point estimates for the odds ratios were almost identical, although the confidence intervals were wider, as expected with a smaller sample size.

## Discussion

To the authors’ knowledge, this is the first study to suggest a benefit of preventing recrudescent compared to new infections. As expected, fever was more common among children with malaria infection than among those without. However, this study demonstrated that recrudescent infections are associated with worsened haematological outcomes compared to a new malaria infection acquired after successful treatment. Also, recrudescent infections occur sooner after treatment, leading to an overall increase in the incidence of infections. Thus, although new and recrudescent infections are clinically indistinguishable, curing the initial infection, even if a new infection occurs, improves the overall health of the patient.

Impaired haematological recovery in children with recrudescent parasitaemia is an objective measure and essential to the overall health of the population. With persistent infection, even when it is microscopically undetectable, on-going inflammation and erythrocyte destruction interferes with the recovery of the red blood cells [6]. Interestingly, in almost all cases, haemoglobin levels increased despite the failure to completely cure the infection.

In this study, age was not a risk factor for recurrent infection in general nor was it associated with the classification of new versus recrudescent infection. This may be due to the intensity of transmission at the study site leading to early acquisition of immunity or it may be attributed to the homogeneity in the age of the participants.

As expected, infections were polyclonal within individuals in this study. At baseline measurement, patients with higher MOI at the time of recurrence were more likely to be classified as having a recrudescent infection. This may be a true biological phenomenon or it may be due to chance. When a participant’s MOI at baseline or at the time of reinfection was high, new infections could have been misclassified as recrudescent infections because some alleles from a new parasite genotype may have matched between the original and recurrent infection by chance. Infection with multiple malaria parasite strains may have had a greater probability of containing one or more malaria strains that were resistant to treatment, leading to a recrudescent infection.

Any genotyping technique may misclassify infections. If a parasite genotype is present, but below the level of detection at the initial infection and then grows to detectable levels at the time of recurrent infection, it may be classified as a new infection even though it was present initially. Alleles were identified using capillary electrophoresis, a method that is highly sensitive and less likely than other genotyping methods to fail to detect minority populations [7]. Another limitation was the inability to discern haplotypes. Consequently, polyclonal infections that shared the same collection of alleles between the initial and recurrent infections may have appeared to be recrudescent infections, even though the haplotype profiles for the parasites in the recurrent infection were different.

The most notable limitation of this study is that these SP efficacy results may not be applicable to the currently used artemisinin-based combination therapies. SP was a failing drug at the time of this study, which resulted in more recrudescent infections than would be expected in a current artemisinin combination therapy drug efficacy study. It is also possible that current, more effective drugs keep recrudescent infections at a lower density of parasitaemia, which may produce a different clinical presentation compared to patients treated with SP. In addition, in current protocols for assessment of anti-malarial drug efficacy, all episodes of recurrent parasitemia are treated with rescue therapy, regardless of symptoms. As a result, this experiment is unlikely to be repeated.

In high transmission settings, when new infections frequently interfere with assessment of a drug’s efficacy in clearing the initial infection, using molecular methods to distinguish new from recrudescence provides useful information. This study’s results indicate that treating clinical malaria with drugs that provide sterile cure is the most beneficial to the patient when compared to suppressive treatment that fails to eliminate all asexual parasites from the blood even while preventing subsequent infections.

The results of this study confirm the paramount importance of providing effective treatment of all malaria infections. If malaria infection occurs several weeks after the initial treatment, full parasitological cure followed by a new infection is better for patients than suppressive treatment with persistent sub-patent infection that reaches detectable levels again. Thus, anti-malarial drugs that provide prolonged prophylaxis but poor curative efficacy will have a long-term detrimental effect on the health of children who suffer from anaemia due to malaria. However, new infections are far from benign. They are just as likely as recurrent ones to cause febrile illnesses in children, prompting treatment-seeking behaviour and leading to further burden on the health care system.

## Conclusion

In areas of continuous exposure to malaria, distinguishing new from recrudescent infections in cases of recurrent parasitaemia after treatment has important clinical implications. Although new and recrudescent infections cause febrile illnesses at a similar rate, recurrence due to recrudescent infection is associated with higher rates of anaemia than new infections and a shorter parasite-free interval. Local decision-makers should take into account the results of genotyping to distinguish new from recrudescent infections when determining treatment policy on a population level. It is appropriate to weigh recrudescent malaria more heavily in assessing treatment efficacy and if two drugs have similar rates of recurrent infections, the one that has higher curative efficacy should be selected over one that allows for more recrudescent infections.

## Competing interests

The authors declare that they have no competing interests.

## Authors’ contributions

AMS, EG, MBL, FKD, MEM, TET, CVP, MKL conceived and designed the study, OMN, PCT, TET, CVP, MKL conducted the clinical study, AMS, EG, LJK, FKD, CGJ conducted the molecular analysis, AMS, EG, MKL conducted the statistical analysis, AMS, EG, MKL wrote the first drafts of the manuscript, MEM, TET, CVP made substantial revisions to the manuscript. All authors reviewed and approved the final manuscript.

## References

[B1] Olliaro P Pinoges L Checchi F Vaillant M Guthmann JP Risk associated with asymptomatic parasitaemia occurring post-antimalarial treatment Trop Med Int Health 2008 13 83 90 10.1111/j.1365-3156.2007.01977.x 18291006

[B2] Plowe CV Kublin JG Dzinjalamala FK Kamwendo DS Chimpeni P Molyneux ME Taylor TE Sustained clinical efficacy of sulfadoxine-pyrimethamine for uncomplicated falciparum malaria in Malawi after 10 years as first line treatment: five year prospective study BMJ 2004 328 545 549 10.1136/bmj.37977.653750.EE 14757706 PMC381042

[B3] Anderson TJ Su XZ Bockarie M Lagog M Day KP Twelve microsatellite markers for characterization of *Plasmodium falciparum* from finger-prick blood samples Parasitology 1999 119 113 125 10.1017/S0031182099004552 10466118

[B4] CVD Malaria Group: Protocols [http://medschool.umaryland.edu/malaria/protocols.asp]

[B5] Greenhouse B Myrick A Dokomajilar C Woo JM Carlson EJ Rosenthal PJ Dorsey G Validation of microsatellite markers for use in genotyping polyclonal *Plasmodium falciparum* infections Am J Trop Med Hyg 2006 75 836 842 17123974 PMC1697796

[B6] Menendez C Fleming AF Alonso PL Malaria-related anaemia Parasitol Today 2000 16 469 476 10.1016/S0169-4758(00)01774-9 11063857

[B7] Nyachieo A Van Omervier C Laurent T Dujardin JC D'Alessandro U *Plasmodium falciparum* genotyping by microsatellites as a method to distinguish between recrudescent and new infections Am J Trop Med Hyg 2005 73 210 213 16014861

